# Phenotypic Diversity in Cell Wall Lignocellulosic Constituents and Ethanol Yield of USDA Guayule and Mariola Germplasm

**DOI:** 10.3390/plants14081239

**Published:** 2025-04-18

**Authors:** Hussein Abdel-Haleem, Steve Masterson, Aaron Sedivy, Rob Mitchell

**Affiliations:** 1 US Arid Land Agricultural Research Center, USDA-ARS, Maricopa, AZ 85138, USA; 2 Wheat, Sorghum and Forage Research Unit, USDA–ARS, University of Nebraska-Lincoln East Campus, Lincoln, NE 68503, USA

**Keywords:** *Parthenium*, guayule, mariola, abiotic stress, lignocellulose, biofuel, ethanol, bioenergy

## Abstract

Guayule *(Parthenium argentatum* A. Gray) is a valuable domestic source for rubber and resin. At its center of origin in the Northern Mexico and Southern Texas deserts, guayule, a perennial shrub, is hybridized with its relative species mariola (*Parthenium incanum* Kunth). As rubber and resin are the main products derived from guayule, there is interest in using guayule bagasse as a bioenergy feedstock to meet the growing bioenergy and biofuel demands. This study aimed to explore and characterize phenotypic diversity in cell wall constituents (lignin, cellulose, and hemicellulose) and their yields among 51 guayule and mariola genotypes under two irrigation regimes (well-watered and water-stressed). Significant genotypic and environmental effects were observed for lignin, cellulose and hemicellulose concentrations, and yields, indicating the wide genetic variability of the collection for bioenergy-related traits. Moderate to high entry-mean heritability values for lignin, cellulose, and hemicellulose suggest that selection is feasible to enhance genetic gain. Significant positive correlations were found among cellulose and hemicellulose concentrations and yields, indicating the possibility to select multiple traits together during breeding cycles. High positive correlations between rubber and resin and lignin, cellulose, and hemicellulose yields highlight the opportunity to develop guayule germplasm with enhanced multi-use traits for industrial applications. Wide variations in drought stress indices (stress tolerance index, yield index, and yield stability index) underscore the environmental impact on the lignocellulosic traits. Several genotypes were identified with high stress index scores and could be parental candidates for improving guayule for arid and semi-arid sustainable agricultural systems. The current study is the first to characterize the phenotypic diversities in guayule and mariola for lignocellulosic components and yield, providing the foundation for future breeding efforts aimed at enhancing guayule’s value for diverse production goals and environmental conditions.

## 1. Introduction

Guayule (*Parthenium argentatum* A. Gray) rubber is an alternative domestic natural rubber source for the tire industry [1,2] as well as hypoallergenic latex for medical products [3]. Guayule is a shrub native to the Chihuahuan Desert and is considered a crop candidate for the arid and semi-arid sustainable agricultural systems. As a perennial crop, guayule is harvested after 2–5 years of planting. Guayule bagasse accounts for 85–90% of guayule biomass [4], making guayule a potential biomass feedstock for the emerging bioeconomy in semi-arid regions.

In general, biofuels are produced from feedstocks via two steps: converting feedstock into intermediates using hydrolysis, gasification, hydrothermal liquefication, or pyrolysis [5]; and then synthesizing biofuel from intermediates. Pyrolysis is a technology to deconstruct feedstock at high temperature [6]. Guayule biofuel research has focused primarily on pyrolysis and has been studied extensively [5,7,8,9,10,11]. Due to its unique composition, guayule-derived bio-oil has a higher energy density than bio-oil derived from other biomass resources like wood or grasses [5,8,10]. Luo et al. [12] reported significant genotypic variation in pyrolysis products among guayule genotypes. Deconstruction at low temperature is another technology to break down feedstock using pretreatment followed by hydrolysis [13,14]. This approach is used widely in converting many feedstocks including agricultural residue, wood feedstock, and marine algae [15]. Producing ethanol from guayule biomass has been limited, but a few studies have explained the composition [16] and extraction [17] of guayule lignocellulosic components.

Guayule is a drought-adapted plant that can survive on 250–380 mm of annual rainfall in its native regions [18]; however, its economic productivity is affected by supplemental irrigation level [19,20]. Luo and Abdel-Haleem [21] reported that reducing irrigation amounts resulted in lower biomass, rubber, and resin yields. Their results indicated that the reduction is due to genetic and phenotypic variations among guayule genotypes that include cultivars and advanced germplasm from different gene pools and wild accessions. Luo, Mullen, and Abdel-Haleem [12] demonstrated that guayule cultivars, when planted under different irrigation levels, showed significant genetic variations in pyrolysis coproducts, including non-condensable gases, condensable gases, and bio-char.

Guayule is well suited as a domestic rubber crop in areas of water scarcity or in areas like Texas, New Mexico, Arizona, and South California where irrigation water is severely limited [22]. The strategy is to develop guayule with low water-consuming capabilities while maintaining high rubber, resin, and other co-products under semi-arid conditions. To reach these goals, exploring the genetic diversity of guayule populations and their responses to different irrigation conditions is needed to identify suitable parental candidates for new breeding cycles of crossing and selection to achieve genetic improvement [2,23]. Mariola (*Parthenium incanum* Kunth.) is the closest species to guayule [24]. Mariola has a broad geographic range, occurring from Southwestern Mexico to as far north as Nevada and Utah, USA. This broad geographic range makes mariola a good parental candidate to produce hybrids with favorable characteristics to extend guayule production zones further north. The natural and man-made interspecific hybridizations between guayule and mariola produced fertile hybrids [25,26,27,28]. Natural hybrids between mariola and guayule have positive characteristics from both species. For example, W6 2189 [28] produces more dry biomass than many guayule genotypes [21] and is tolerant to high soil salinity [29].

The main goal of the current study is to explore phenotypic diversities for biofuel-related characteristics and the effects of irrigation levels on the variation of these traits in the USDA guayule and mariola collection. The specific objectives were to: (1) characterize the genetic variations in cellulose, hemicellulose, lignin, and ethanol yield in the USDA guayule and mariola collection; (2) investigate the effects of different irrigation levels (drought abiotic stress) on guayule lignocellulosic production; and (3) study the stability of those components under stress conditions.

## 2. Results and Discussion

### 2.1. Phenotypic Variations in Lignocellulosic Components in Guayule and Mariola Accessions

Guayule bagasse accounts for 85–90% of its total biomass [4], and it is necessary to use that rich source of byproduct to increase guayule farmer revenues via biofuel production. The first step to use guayule as biofuel feedstock is to evaluate the phenotypic diversity of biofuel-related traits in guayule germplasm and collections. As guayule is a perennial crop, it is difficult to repeat its field experiments over time [30,31,32,33,34,35,36]. To overcome that situation, experiments could be conducted over environments [12,19,21,37]. The current research examined the phenotypic variations in USDA guayule and mariola accessions in biofuel-related characteristics and the effect of irrigation levels on the variation of those traits. Mariola (*Parthenium incanum* Kunth.) is the closest species to guayule (*Parthenium argentatum A. Gray*) [24]. Natural hybrids between mariola and guayule have positive characteristics from both: for example, W6 2189 accession [28] produces more dry biomass than many guayule genotypes [21] and is tolerant to high soil salinity [29].

The lignin, cellulose, hemicellulose content and yield, and theoretical ethanol production for the 51 genotypes showed wide phenotypic diversity (Table 1), where the genotypes factor had the highest covariance component (around 50%) compared with environments and their interactions (GxE). For example, the variation in cellulose is controlled by the variation among genotypes (70%), followed by the variation among environments (8%) and then the GxE interaction (4%) (Table 1). The high covariance supports the wide variation in biofuel-related traits and yields in guayule.

Cellulose, hemicellulose, and lignin are the main components in plant cell walls and are critical components in feedstock conversion into biofuel using hydrolysis technology. The concentration of cellulose, hemicellulose, and lignin varies based on plant species and their functions [38]. Previous guayule research demonstrated that cellulose, hemicellulose, and lignin are affected by genotype, environments, and the materials processing degree used in analyses [16,17,39].

It is important to study the phenotypic variation among guayule and its relatives and rank them as a first step to genetically improve guayule as biofuel feedstock. In the current study, cellulose concentration was greater than hemicellulose and lignin across the 51 genotypes (Table 2). Cellulose ranged from 17.86% (PARL 818, a mariola genotype) to 33.85% (PARL 933, a guayule genotype) (Table 2) with an average of 22.5% ± 3.9 for all studied genotypes. In general, mariola genotypes had the highest cellulose concentrations followed by guayule hybrids and then guayule genotypes, with values of 31.9% ± 2.1, 22.8% ± 1.8, and 20.9% ± 1.8, respectively. This indicates that some mariola genotypes could be sources for genes or potential parental lines for hybridization to increase the cellulose content in guayule. Growing conditions affected cellulose content as well, with genotypes grown under less irrigation having lower cellulose content compared with genotypes grown with favorable irrigation (Table 3). For example, cellulose content was reduced by 15% in guayule genotypes that were planted under water stress compared with those planted under no stress (Table 3).

Average hemicellulose concentration varied among mariola genotypes, guayule hybrids, and guayule genotypes, with values of 16.5% ± 0.9, 15.2% ± 1.3, and 15.5% ± 0.8, respectively (Table 2). Among the 51 genotypes, a mariola genotype (PARL 800) was the highest in hemicellulose content with 17.32%, while a guayule hybrid (PI 478,667) was the lowest with 13.57% (Table 2). Under water-stressed conditions, hemicellulose content decreased by 13%, 20%, and 12% in mariola, guayule hybrids, and guayule genotypes, respectively (Table 3). The third component lignin averaged 16.3% ± 1.6 for the 51 studied genotypes (Table 2).

Lignin concentration ranged from 12.30% (PI 478,667, guayule hybrid) to 21.31% (PI 478,654, guayule). Mariola genotypes averaged 16.1% ± 1.0, guayule genotypes averaged 16.6% ± 1.5, and guayule hybrids averaged 14.8% ± 1.9 (Table 2). Lignin increased in mariola and guayule groups grown under water-stressed conditions by 2.72% and 2.2%, respectively, compared with those grown under no-stress conditions. No-stress conditions increased lignin in guayule hybrids (Table 3). Several studies found that lignin increased in response to drought stress [40,41,42,43]. This suggests that lignin could play a role in drought stress tolerance in guayule and mariola, and that effect is dependent on genotype as well as origin. For example, relative lignin content (less irrigation–normal irrigation) reached its highest in the PARL 818 mariola genotype that was collected from the Texas desert, as well as wild guayule genotypes W6 2244 and W6 2245, collected from Durango, Mexico, and W6 2248, collected from Zacatecas, Mexico (Supplementary Tables S1 and S2).

Crop biomass is an important factor in biofuel feedstocks. Besides the lignocellulosic contents, biomass determines the final yield of biofuel products. The lignocellulosic components yield was estimated based on guayule dry biomass to explore the variations in the yield of those components (Table 2). Even though cellulose yield averaged 5233 ± 1765 kg ha^−1^ for the 51 genotypes, guayule hybrids had higher cellulose yield followed by mariola and then guayule genotypes with 7434 ± 2096 kg ha^−1^, 6830 ± 1473 kg ha^−1^, and 4498 ± 1056 kg ha^−1^, respectively (Table 2). The same trend was observed in hemicellulose and lignin, where guayule hybrids had higher yields than guayule genotypes (Table 2). For example, the hemicellulose average for guayule hybrids was 4834 ± 1526 kg ha^−1^ and 2942 ± 749 kg ha^−1^ for guayule genotypes (Table 2). These data revealed greater yields for lignocellulosic components in guayule hybrids (hybridization with mariola or other species) were larger than guayule genotypes.

Hybridization between guayule and its relatives resulted in increased progeny biomass [44,45,46]. For example, PI 478,666 (hybrid of guayule × *P. tomentosum* [47]), PI 478,667 (hybrid of guayule × *P. fruticosum* [47]), and W6 551 (hybrid of guayule × *P. tomentosum* [48]) produced 5667 kg ha^−1^, 6464 kg ha^−1^, and 10,437 kg ha^−1^ cellulose compared with the guayule genotypes group average of 5233 kg ha^−1^ (ranging from 7827 kg ha^−1^ to 2513 kg ha^−1^). Still, the hybrid of guayule and mariola, W6 2189, produced high cellulose yield (6218 kg ha^−1^) compared with guayule genotypes (Table 2). This demonstrates the potential to use guayule relatives to improve the biofuel characteristics of guayule.

Dry biomass was affected by less irrigation [21], which is reflected in the lignocellulosic components yield of the current study. For example, the cellulose yields in guayule hybrids were 11,415 kg ha^−1^ and 2615 kg ha^−1^ under normal and reduced irrigations, respectively (Tabel 3). Furthermore, the cellulose yields of guayule genotypes were 6412 kg ha^−1^ and 1608 kg ha^−1^ under non-stressed and stressed conditions, respectively. Cellulose yields in mariola genotypes accounted for 9960 kg ha^−1^ and 3177 kg ha^−1^ under non-stressed and stressed conditions, respectively. Mariola genotypes were affected less than guayules and guayule hybrids (Table 3). These data suggest that selection for old hybrids was based on visual growth and appearances, and future selection based on biofuel traits could develop genotypes with superior characteristics, of both guayule and its relative, in lignocellulose traits and yield under stress conditions.

The theoretical ethanol yield showed a wide range among the 51 tested genotypes (3084 L ha^−1^ to 10,982 L ha^−1^) with an average of 5342 ± 1720 L ha^−1^ (Table 2). The wide range of phenotypic ethanol yield could indicate the wide genetic diversity that controls ethanol yield, and thus possibilities to genetically improve this trait. The wide range was observed in all three classes, with guayule hybrids expressing the widest range (10,982 L ha^−1^ produced by PI 599,675 to 4901 L ha^−1^ produced by PI 478657) and an average of 7578 ± 2225 L ha^−1^. The average ethanol yield for guayule genotypes and mariola genotypes were 4716 ± 1138 L ha^−1^ and 6223 ± 1214 L ha^−1^, respectively (Table 2). As observed with other traits, water stress conditions (less irrigations) reduced the ethanol production by 68%, 77%, and 75% in mariola genotypes, guayule hybrids, and guayule genotypes, respectively (Table 3).

### 2.2. Heritability of Parthenium Biofuel-Related Traits

**Figure 1:**
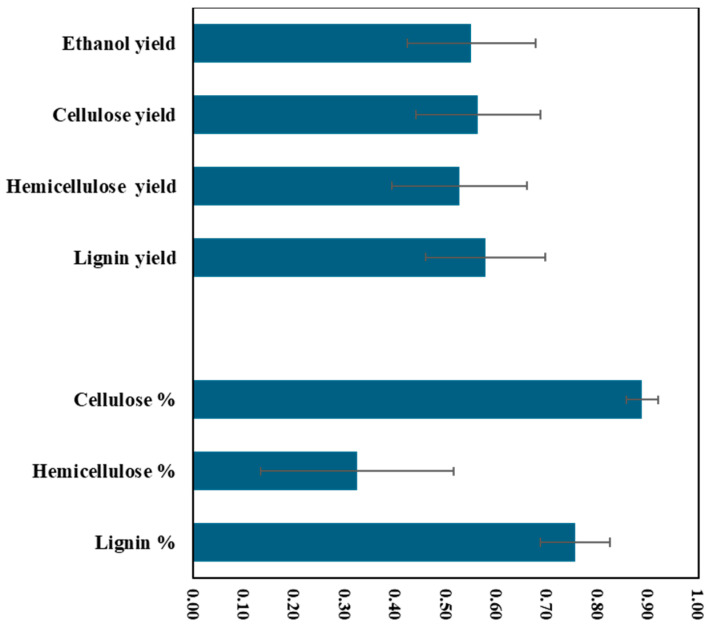
Broad-sense heritability values (h^2^) of bioenergy-related traits in 51 *Parthenium* genotypes from the USDA germplasm collection.

The high heritability estimates indicate the feasibility of selection for traits of interest during the early generations of the breeding cycle [49]. The current study had high broad-sense heritability estimates for lignin (h^2^ = 0.76) and cellulose (h^2^ = 0.89), while the estimates were low for hemicellulose (h^2^ = 0.32) (Figure 1). The low heritability estimates in traits such as hemicellulose indicate a high level of environmental effects. Under such conditions, it is important to test guayule genotypes under several environments and/or growing conditions. The heritability estimates suggest a high level of genetic control of the lignocellulosic components in guayule. The results are in agreement with the heritability estimates for lignocellulosic components of maize (*Zea mays* L.) [50], rape (*Brassica napus* L.) [51] and rice (*Oryza sativa* L.) [52]. When dry biomass was considered, the heritability estimates for lignocellulosic components yield were reduced to moderate levels of heritability (Figure 1). The heritability values ranged from h^2^ = 0.53 (hemicellulose yield) to h^2^ = 0.58 (lignin yield) (Figure 1). This could be explained by the variable environmental effects of irrigation on the inheritance of these traits and the need for exploring more exotic germplasm. The heritability estimates for ethanol yield were 0.55. These findings suggest that these guayule lignocellulosic traits are heritable and could be modified through selection during early generations of breeding programs.

### 2.3. Stress Indices of Parthenium Genotypes Under Water Stress Conditions

Understanding lignocellulosic components yield stability for guayule and mariola genotypes under different irrigation conditions can be used to increase the genetic gains of guayule breeding programs targeting less water requirements in semi-arid zones (Table 4). To understand the complicity of the stability concept and avoid overestimating the relation between yield traits and stability indices, eight different stability indices were estimated for the studied traits. The current results indicated that guayule and mariola genotypes exhibited a wide range of stability indexes (Supplementary Table S3), suggesting that the USDA germplasm collection of guayule and mariola genotypes has drought-tolerant and drought-susceptible genotypes based on the lignocellulosic traits. Among the indices used to measure the stability of yield traits under stress are stress tolerance index (STI), yield index (YI), and yield stability index (YSI) (Table 4). As lignocelluloses and ethanol yields are the final products, the stability of yield traits under stress is a crucial criterion for identifying stable advanced germplasm grown under different environments. Identifying such an advanced germplasm using those indices is a valuable tool to increase the genetic gains of these traits. A high STI score indicates that a genotype is drought stress-tolerant, a high YI score indicates yield suitability of a genotype grown under stress conditions, and a high YSI score indicates the stability of a genotype to yield under stress and non-stress conditions [53]. Because these indices vary in their calculation methods, their ability to detect variation among the studied genotypes also varies. For example, the range of YSI for lignin yield was 0.10–0.53, while STI and YI ranged from 0.07–1.16 and 0.07–2.16, respectively (Table 4), indicating that in the current study, YI can detect a wider range of variations and could serve as a reliable indicator of yield stability.

PARL 800 and PARL 818 are considered drought-tolerant genotypes with greater STI, YI, and YSI than PARL 792, a drought-susceptible genotype (Table 4). The PARL 818 accumulated lignin under stress conditions, while lignin concentration for PARL 800 was reduced under stress conditions (Supplementary Table S2).

PARL 818 had higher values than PARL 800 for stress tolerance (TOL) and stress tolerance efficiency (STE) and relative stress index (RSI) for lignin (Supplementary Table S3). This could indicate that the drought tolerance mechanisms in those genotypes are different, and lignin content could play a role in the drought resistance of PARL 818. Among guayule hybrids, W6 2271, a wild genotype collected from Coahuila, Mexico, is considered drought-tolerant due to the high values of STI, YI, and YSI (Table 4). PI 599,675 and PI 599,676 had high STI and YI scores and low YSI scores, indicating that those genotypes are drought-tolerant but unstable under stress conditions (Table 3). These findings suggest that drought tolerance gene(s) could be different than yield stability genes in guayule. Guayule W6 7157, a cultivar developed for California growing conditions, had high values of STI, YI, and YSI. In general, guayule cultivars developed for California (PI 478,665) and/or Arizona (PI 599,674, PI 599,677, PI 599,678, and PI 599,679) tended to have high YSI values and low YI and STI values (Table 4, Supplementary Table S1), indicating the stability of those genotypes under similarly tested environments. These results support the fact that guayule and mariola have a wide range of drought tolerance genotypes that are stable under different growing environments, and those genotypes have different drought tolerance mechanisms based on their origin and development.

### 2.4. Correlation Among Economic Traits

To understand the correlations among bioenergy-related traits in guayule, correlation analyses were conducted for traits related to plant biomass and lignocellulosic components (Table 5). In general, the low correlation coefficient indicates the independence of traits. There were significant correlations between lignin and hemicellulose contents (r = 0.365, *p* < 0.001) and hemicellulose and cellulose contents (r = 0.492, *p* < 0.0001).

Dry biomass was negatively correlated with lignin and positively correlated with cellulose (Tabel 5). Unsurprisingly, hemicellulose and cellulose concentrations were positively correlated with hemicellulose and cellulose yields (Table 5). Even though lignin content correlated negatively with dry biomass (r = −0.267, *p =* 0.002), dry biomass correlated positively with lignin yield (r = 0.977, *p* < 0.0001), indicating that selecting for lignin yield could be the target instead of lignin content. Lignin yield also correlated with cellulose and hemicellulose yields (Table 5). These fundings suggest the possibility of selecting multiple lignocellulosic traits at once to improve their content and/or yield. Theoretical ethanol yield correlated negatively with lignin (r= −0.364, *p* = 0.002) and positively with cellulose (r = 0.522, *p* < 0.0001) and hemicellulose (r = 0.175, *p* = 0.043). There were highly significant correlations between ethanol yield and dry biomass, lignin yield, cellulose yield, and hemicellulose yield (Table 5), indicating that selection for lignocellulose yields will result in increased ethanol yield.

As rubber and resin are the main products of guayule, the current study compared those products with lignocellulosic components (Table 5). Both rubber and resin contents were positively correlated with lignin content and negatively correlated with cellulose and hemicellulose. This could be related to cell function and responses to environmental stresses. In contrast, rubber and resin yields had highly significant positive correlations with lignocellulosic yields and ethanol production (Table 5). These significant correlations suggest that selection for increased lignocellulosic components yields will lead to increases in rubber and resin yields as well, thus resulting in the development and release of advanced germplasm for multiple industrial uses.

## 3. Materials and Methods

### 3.1. Plant Materials and Experiment Layout

A set of 51 Parthenium genotypes (37 guayule, 8 guayule hybrid, and 6 mariola genotypes) were evaluated (Supplementary Table S1). Seeds were accessed from the USDA *Parthenium* germplasm collection. Seeds of each genotype were planted in the greenhouse at 13 August 2019, then transplanted to the field as seedlings during the fourth week of October 2019. Healthy seedings were transplanted to two-row field plots in two trials varied in irrigation levels (will be referred to as environments). The length of the plots was 3 m long, the distance between each row within each plot was 1 m, and the distance between plants within the row was 0.3 m. The two irrigation treatments (water-stressed and well-irrigated) were conducted at Maricopa Agricultural Center, University of Arizona, Maricopa, AZ (33°03′58″ N 111°58′31″ W). The soil at the trial location was a Casa Grande series (fine-loamy, mixed, hyperthermic Typic Natrargids). For each irrigation treatment, genotypes and checks were arranged in an augmented block design [54,55,56]. The six guayule check genotypes were randomized in each of the four blocks with four replications for each irrigation treatment. To reach a suitable stress level, plots in both well-irrigated and water-stressed trials were furrow irrigated bi-weekly until plants were established; then, irrigation was withheld in the water-stressed trial for three months, while the well-irrigated trial was irrigated every 2–3 weeks based on weather conditions. The plants were harvested around the first week of March 2022 by hand at 5 cm above the soil level from each plot. The harvested plants were dried, chipped, finely ground, and stored following the protocols and equipment mentioned in Luo and Abdel-Haleem [21]. Guayule biomass (kg ha^−1^) based on dry weight was determined from the dried harvested plants.

### 3.2. Lignocellulosic, Rubber, and Resin Analysis

Ground samples were used to determine acid detergent fiber (ADF) and neutral detergent fiber (NDF) using the Ankom method [57]. Acid detergent lignin (ADL) was determined by placing 24 oven-dried post-ADF sample bags in an acid-resistant container containing 300 mL of 72% sulfuric acid for 3 h at 22 °C, with gentle agitation every 30 min. After the acid treatment, the acid was decanted and sample bags were placed in a 4 L glass jar and flushed with tap water for 30 min. After the water rinse, samples were dried overnight in a 100 °C oven and reweighed. Hemicellulose and cellulose were calculated according to Hindrichsen et al. [58]. Theoretical ethanol yield was calculated according to Abideen et al. [59]. Ground samples were used to determine the resin and rubber contents using an NIR DA7250 At-line NIR Analyzer (PerkinElmer, Shelton, CT, USA).

### 3.3. Statistical Analysis

An analysis of variance (ANOVA) of the studied traits across genotypes and irrigation levels was conducted by SAS PROC MIXED 9.4 software using restricted maximum likelihood (REML) with irrigation levels (refer as environments),, genotypes, block (environment), check x environment, and genotype x environment being considered random effects (Statistical Analysis System, SAS Institute Inc., Cary, NC, USA, 1989–2023). For each studied trait, best linear unbiased predictors (BLUPs) for each genotype was estimated using SAS PROC MIXED software. Pearson’s phenotypic correlation coefficient analyses were conducted to assay the relationships among studied traits. The broad-sense heritability based on the entry-mean was calculated as: h^2^ = σ^2^_G_/(σ^2^_G_ + σ^2^_G × E/_e), where σ^2^_G_ is genetic variance among genotypes, σ^2^_G × E_ is genotype x environment interaction (GxE) variance, and *e* represents the number of environments [49,60]. Stress tolerance index (STI)  =  (y_C_ × y_D_)/Y_C_^2^ [61], yield index (YI)  =  y_D_/Y_D_ [62], yield stability index (YSI)  =  y_D_/y_C_ [53], stress tolerance (TOL)  =  y_C_ − y_D_ [63], relative stress index (RSI)  =  (y_C_/y_D_)/(Y_C_/Y_D_) [64], stress tolerance efficiency (STE) = (y_D_/y_C_) × 100 [64], geometric mean productivity (GMP)  =  √(y_D_ × y_C_) [61], and harmonic mean (HM)  =  2(y_C_ × y_D_)/(y_C_ + y_D_ ) [65] were calculated, where y_C_ and y_D_ are the yield of a genotype under well-watered irrigation (C) and stressed irrigation (D), respectively, while Y_D_ is the yield mean under drought stress (stressed irrigation) conditions.

## 4. Conclusions

Rubber and resin are the main products from the domestic rubber plant, guayule. The current study was aimed at identifying components from guayule that could be used as intermediate chemicals for the emerging bioeconomy. To achieve that target, phenotypic diversity in lignocellulosic components in the USDA guayule and mariola collections were explored. The collections included improved germplasm and cultivars and wild accessions collected from natural habitats in Mexico and the United States. Wide ranges in compositional components and yield indicate the high phenotypic diversity in the studied traits, reflecting different origins, adaptations, and genetic makeups of those genotypes. Several genotypes, such as PARL 818, W6 2272, and W6 7157, were identified with high yield and lignocellulosic components stability when grown under contrasting environments and high responses to drought stress (less irrigation) conditions. Those genotypes could be parental materials to improve bioenergy-related traits in guayule. High heritability estimates and significant positive correlations among the studied lignocellulosic traits indicated the high possibilities to breed/select for more than one trait during early generations of the breeding process. High positive correlations between lignocellulosic traits and rubber and resin suggest that combining both traits in improved germplasm is feasible. These findings lay the foundation for guayule breeding efforts to select parental candidates for breeding programs to grow guayule under different growing conditions and to achieve multiple production goals.

## Figures and Tables

**Table 1:** Covariance parameter estimates of the MIXED model for biofuel-related traits of 51 *Parthenium* genotypes.

	Lignin	Hemicellulose	Cellulose	Lignin Yield	Hemicellulose Yield	Cellulose Yield	Ethanol Yield
Environments (E)	0.00	0.00	1.75	1,725,314	2,089,594	5,163,086	5,099,719
Genotypes (G)	1.69	0.46	15.52	7,989,112	3,879,290	545,155	1,625,688
GXE	0.00	0.11	0.94	0	0	0	0

**Table 2:** Best linear unbiased predictions (BLUPs) of 51 *Parthenium* genotypes for biofuel production traits.

Genotype	Lignin	Hemicellulose	Cellulose	Lignin Yield	Hemicellulose Yield	Cellulose Yield	Ethanol Yield
	%	%	%	kg ha^−1^	kg ha^−1^	kg ha^−1^	L ha^−1^
Mariola							
PARL 792	16.57	15.84	28.66	3131	3026	5418	5191
PARL 798	17.09	15.2	29.87	2627	2385	4618	4288
PARL 799	15.84	16.78	33.46	4008	4305	8194	7363
PARL 800	16.23	17.32	33.25	3730	3862	7212	6595
PARL 815	16.3	16.33	32.29	3806	3910	7459	6758
PARL 818	14.43	17.28	33.85	3643	4282	8082	7143
Guayule hybrid							
PI 478,657	17.12	15.4	21.34	3268	3024	4644	4901
PI 599,675	14.86	14.89	21.39	6874	7288	10,248	10,982
PI 599,676	14.87	14.11	22.36	4867	4785	7731	7841
PI 478,666	12.58	15.54	22.58	2987	3767	5667	5595
PI 478,667	12.3	13.57	24.15	3185	3651	6464	5999
W6 551	13.83	16.77	25.64	5506	6637	10,437	10,113
W6 2271	15.5	14.03	20.32	5897	5538	8069	8822
W6 2189	17.46	17.21	24.57	4085	3985	6218	6371
Guayule							
W6 2272	15.63	15.29	20.68	4063	4040	5640	6201
W6 2196	15	13.81	18.85	2779	2656	4012	4290
W6 2244	15.97	16.05	20.78	2508	2454	3946	4025
W6 2245	16.24	15.1	19.82	2021	1852	3240	3197
W6 2248	15.94	16.89	24.69	3631	3874	6065	6079
W6 2260	17.25	16.03	22.02	3348	3154	5004	5163
PARL 805	16.4	15.46	20.46	3045	2881	4215	4573
PARL 816	16.51	15.03	20.7	2504	2310	3845	3907
PARL 820	14.87	13.93	19.83	2847	2704	4409	4515
PI 478,639	18.01	15.98	23.38	2928	2696	4484	4528
PI 478,640	16.92	16	22.08	2593	2466	3776	3989
PI 478,642	18.48	15.5	19.36	2511	2212	3597	3726
PI 478,643	17.35	16.41	23.24	5450	5407	7827	8379
PI 478,644	14.97	14.41	19.16	2760	2658	4076	4273
PI 478,649	16.89	15.67	18.67	2950	2818	4235	4509
PI 478,653	17.96	14.83	18.37	2874	2492	3741	4088
PI 478,654	21.31	16.23	21.99	3212	2670	4379	4590
PI 478,655	16.71	16.11	21.43	3462	3360	4985	5290
PI 478,656	16.93	15.44	19.38	2650	2440	3861	4027
PI 478,659	15.29	14.92	20.84	2608	2480	4066	4120
PI 478,665	13.27	15.94	23.75	2480	3031	4638	4573
W6 7157	15.63	15.29	20.68	4063	4040	5640	6201
PI 599,674	15.78	14.62	20.1	3406	3221	5076	5281
PI 599,677	15.79	15.23	19.73	2934	2708	3538	4157
PI 599,678	17.16	16.97	19.35	2206	2210	2513	3121
PI 599,679	15.88	15.57	20.37	3116	3078	4155	4663
PARL 912	18.97	15.34	19.87	2697	2307	3838	3968
PARL 917	18.16	16.68	22.7	3936	3728	5665	5997
PARL 920	16.16	14.97	20.89	2406	2235	3659	3084
PARL 922	16.68	15.83	21.42	4106	3929	5932	6285
PARL 924	15.39	16.38	23.25	2840	2873	4634	4643
PARL 927	15.15	14.96	19.74	2516	2441	3933	4005
PARL 929	15.4	14.87	18.3	2694	2592	3848	4112
PARL 930	18	15.59	23.99	3082	2782	4550	4670
PARL 931	17.96	16.13	22.88	4937	4744	7178	7560
PARL 932	17.55	15.89	22.19	2898	2685	4232	4408
PARL 933	15.62	13.69	17.86	2866	2653	4016	4299

**Table 3:** Best linear unbiased predictions (BLUPs) for *Parthenium* genotypes and hybrids growing under water-stressed (DRY) and well-watered (IRRI) conditions.

	Lignin %		Hemicellulose %		Cellulose %			
	IRRI	Dry	IRRI	Dry	IRRI	Dry		
Mariola	15.84	16.28	17.55	15.22	33.99	29.27		
Guayule hybrid	15.06	14.43	16.75	13.47	25.87	19.14		
Guayule	16.34	16.70	16.34	14.46	22.22	18.73		
	Lignin yield kg ha^−1^		Hemicellulose yield kg ha^−1^		Cellulose yield kg ha^−1^		Ethanol yield L ha^−1^	
	IRRI	Dry	IRRI	Dry	IRRI	Dry	IRRI	Dry
Mariola	5176.6	1607.1	5636	1384	9960	3177	9254	2757
Guayule hybrid	6876.9	1928.7	7592	1717	11,415	2615	11,318	2854
Guayule	4351.6	1390.5	4461	1020	6412	1608	6833	1823

**Table 4:** Yield stability index (YSI), yield index (YI), and stress tolerance index (STI) estimates for 51 *Parthenium* genotypes for lignin yield (ADL), hemicellulose yield (Hem), cellulose yield (Cell), and ethanol yield (ETO).

	YSI				YI				STI			
	ADL	Hem	Cell	ETO	ADL	Hem	Cell	ETO	ADL	Hem	Cell	ETO
Mariola												
PARL 792	0.21	0.13	0.22	0.20	0.73	0.60	0.98	0.81	0.24	0.15	0.29	0.24
PARL 798	0.33	0.21	0.35	0.31	0.82	0.68	1.14	0.91	0.20	0.12	0.24	0.19
PARL 799	0.35	0.28	0.35	0.33	1.39	1.61	2.19	1.76	0.53	0.49	0.89	0.66
PARL 800	0.50	0.46	0.55	0.51	1.51	1.90	2.32	1.91	0.44	0.41	0.64	0.51
PARL 815	0.12	0.05	0.11	0.10	0.52	0.32	0.74	0.56	0.22	0.10	0.32	0.22
PARL 818	0.42	0.37	0.41	0.40	1.45	1.98	2.40	1.96	0.48	0.56	0.92	0.68
Guayule hybrid												
PI 478,657	0.35	0.22	0.25	0.27	1.09	0.89	0.88	0.95	0.33	0.19	0.20	0.24
PI 599,675	0.21	0.16	0.19	0.19	1.50	1.61	1.58	1.57	1.04	0.88	0.85	0.94
PI 599,676	0.34	0.28	0.30	0.31	1.48	1.58	1.55	1.55	0.61	0.48	0.53	0.56
PI 478,666	0.10	0.03	0.08	0.08	0.40	0.18	0.51	0.42	0.15	0.06	0.20	0.16
PI 478,667	0.33	0.30	0.26	0.39	1.02	1.54	1.24	1.25	0.30	0.42	0.39	0.28
W6 551	0.33	0.29	0.26	0.29	1.70	2.45	2.07	2.04	0.85	1.09	1.08	1.03
W6 2271	0.42	0.40	0.35	0.39	2.24	2.61	2.06	2.27	1.16	0.90	0.79	0.95
W6 2189	0.19	0.15	0.16	0.17	0.85	0.87	0.85	0.86	0.36	0.27	0.29	0.31
Guayule												
W6 2272	0.48	0.48	0.45	0.47	1.75	2.25	1.80	1.90	0.61	0.55	0.47	0.55
W6 2196	0.12	0.02	0.08	0.08	0.39	0.08	0.31	0.30	0.12	0.02	0.08	0.08
W6 2244	0.33	0.20	0.24	0.26	0.72	0.60	0.64	0.66	0.15	0.10	0.11	0.12
W6 2245	0.19	0.03	0.12	0.11	0.37	0.07	0.27	0.25	0.07	0.01	0.04	0.04
W6 2248	0.35	0.26	0.26	0.29	1.21	1.33	1.21	1.25	0.40	0.36	0.37	0.39
W6 2260	0.41	0.28	0.26	0.31	1.12	1.01	0.84	0.98	0.29	0.20	0.18	0.22
PARL 805	0.36	0.27	0.24	0.28	1.08	1.03	0.81	0.95	0.31	0.21	0.18	0.23
PARL 816	0.36	0.24	0.31	0.31	0.78	0.68	0.78	0.76	0.17	0.10	0.13	0.14
PARL 820	0.23	0.14	0.21	0.20	0.63	0.50	0.65	0.63	0.16	0.09	0.13	0.14
PI 478,639	0.31	0.14	0.19	0.21	0.83	0.51	0.61	0.65	0.22	0.10	0.13	0.15
PI 478,640	0.25	0.15	0.21	0.22	0.69	0.56	0.66	0.66	0.19	0.11	0.14	0.14
PI 478,642	0.23	0.09	0.13	0.15	0.55	0.26	0.34	0.39	0.13	0.04	0.06	0.07
PI 478,643	0.37	0.26	0.24	0.28	1.96	1.88	1.53	1.74	1.00	0.72	0.64	0.77
PI 478,644	0.27	0.17	0.21	0.22	0.75	0.62	0.67	0.70	0.20	0.12	0.14	0.16
PI 478,649	0.25	0.11	0.14	0.16	0.69	0.43	0.43	0.52	0.18	0.09	0.09	0.12
PI 478,653	0.34	0.23	0.24	0.27	0.94	0.76	0.67	0.79	0.25	0.13	0.13	0.16
PI 478,654	0.27	0.13	0.19	0.20	0.78	0.45	0.55	0.60	0.21	0.08	0.11	0.13
PI 478,655	0.29	0.22	0.23	0.25	1.00	0.99	0.91	0.96	0.33	0.24	0.23	0.27
PI 478,656	0.30	0.16	0.19	0.21	0.73	0.51	0.50	0.58	0.17	0.09	0.09	0.11
PI 478,659	0.31	0.21	0.27	0.27	0.74	0.68	0.74	0.73	0.17	0.11	0.14	0.14
PI 478,665	0.53	0.47	0.40	0.45	1.14	1.67	1.36	1.37	0.24	0.31	0.30	0.29
W6 7157	0.48	0.48	0.45	0.47	1.75	2.25	1.80	1.90	0.61	0.55	0.47	0.55
PI 599,674	0.42	0.35	0.36	0.37	1.14	1.18	1.07	1.12	0.29	0.22	0.21	0.24
PI 599,677	0.48	0.54	0.55	0.53	1.25	1.62	1.30	1.37	0.32	0.26	0.20	0.26
PI 599,678	0.51	0.50	0.49	0.50	0.99	1.26	0.85	1.00	0.19	0.17	0.10	0.14
PI 599,679	0.47	0.46	0.41	0.45	1.32	1.65	1.22	1.36	0.35	0.31	0.24	0.30
PARL 912	0.19	0.06	0.16	0.14	0.47	0.20	0.39	0.37	0.11	0.03	0.06	0.07
PARL 917	0.38	0.30	0.30	0.33	1.32	1.34	1.16	1.25	0.44	0.31	0.29	0.35
PARL 920	0.16	0.06	0.15	0.09	0.43	0.21	0.43	0.27	0.11	0.04	0.08	0.06
PARL 922	0.32	0.29	0.30	0.31	1.15	1.30	1.15	1.19	0.40	0.31	0.29	0.33
PARL 924	0.25	0.17	0.22	0.21	0.67	0.65	0.73	0.70	0.18	0.13	0.16	0.16
PARL 927	0.26	0.11	0.17	0.18	0.62	0.38	0.47	0.50	0.14	0.07	0.09	0.10
PARL 929	0.37	0.23	0.24	0.28	0.92	0.78	0.69	0.79	0.22	0.14	0.13	0.16
PARL 930	0.24	0.13	0.19	0.19	0.76	0.50	0.68	0.66	0.23	0.10	0.16	0.16
PARL 931	0.21	0.12	0.16	0.16	1.01	0.78	0.84	0.88	0.47	0.27	0.30	0.34
PARL 932	0.29	0.19	0.27	0.25	0.83	0.71	0.84	0.81	0.23	0.14	0.17	0.18
PARL 933	0.30	0.14	0.19	0.21	0.84	0.54	0.59	0.67	0.23	0.11	0.12	0.16

**Table 5:** Phenotypic correlation coefficients (top line) and significant level (bottom line) among bioenergy, rubber, and resin traits for 51 Parthenium genotypes from the USDA germplasm collection.

	Resin	Rubber	ADL	Hem	Cell	DWT	Res YD	RubberYD	ADL YD	Hem YD	Cell YD
Rubber %	0.581										
	*<0.0001*										
Lignin % (ADL)	0.254	0.412									
	*0.003*	*<0.0001*									
Hemicellulose % (Hem)	−0.244	−0.064	0.365								
	*0.004*	*0.462*	*<0.0001*								
Cellulose % (Cell)	−0.555	−0.759	−0.157	0.492							
	*<0.0001*	*<0.0001*	*0.069*	*<0.0001*							
Dry weight (kg·ha^−1^) (DWT)	0.054	−0.238	−0.267	0.055	0.373						
	*0.541*	*0.006*	*0.002*	*0.526*	*<0.0001*						
Resin yield (kg·ha^−1^) (Res_YD)	0.375	−0.017	−0.145	−0.058	0.143	0.928					
	*<0.0001*	*0.843*	*0.097*	*0.506*	*0.100*	*<0.0001*					
Rubber yield (kg·ha^−1^) (Rubr_YD)	0.494	0.429	0.032	−0.066	−0.198	0.708	0.854				
	*<0.0001*	*<0.0001*	*0.712*	*0.449*	*0.022*	*<0.0001*	*<0.0001*				
Lignin yield (kg·ha^−1^) (ADL_YD)	0.131	−0.162	−0.087	0.109	0.346	0.977	0.948	0.762			
	*0.132*	*0.063*	*0.317*	*0.208*	*<0.0001*	*<0.0001*	*<0.0001*	*<0.0001*			
Hemicellulose yield (kg·ha^−1^) (Hem_YD)	0.010	−0.253	−0.231	0.168	0.429	0.992	0.902	0.685	0.974		
	*0.907*	*0.003*	*0.007*	*0.052*	*<0.0001*	*<0.0001*	*<0.0001*	*<0.0001*	*<0.0001*		
Cellulose yield (kg·ha^−1^) (Cell_YD)	−0.116	−0.401	−0.281	0.177	0.575	0.966	0.825	0.538	0.931	0.975	
	*0.183*	*<0.0001*	*0.001*	*0.041*	*<0.0001*	*<0.0001*	*<0.0001*	*<0.0001*	*<0.0001*	*<0.0001*	
Ethanol yield (kg·ha^−1^)	−0.068	−0.346	−0.264	0.175	0.522	0.981	0.860	0.598	0.954	0.991	0.996
	*0.438*	*<0.0001*	*0.002*	*0.043*	*<0.0001*	*<0.0001*	*<0.0001*	*<0.0001*	*<0.0001*	*<0.0001*	*<0.0001*

## Data Availability

All data generated or analyzed during this study are available upon request to corresponding author (Hussein Abdel-Haleem) at hussein.abdel-haleem@usda.gov.

## Supplementary Materials

The following supporting information can be downloaded at: https://www.mdpi.com/article/10.3390/plants14081239/s1, Table S1: Origin and breeding status of the 51 Parthenium genotypes used in the current study. Table S2: BLUP estimates for 51 Parthenium genotypes for traits related to bioenergy production planted under water stress (dry) and non-stress (IRRI) conditions. Table S3: Stability indices estimate of 52 Parthenium genotypes for traits related to bioenergy production planted under stress and non-stress conditions.
